# Synovial fluid IL-1β appears useful for the diagnosis of chronic periprosthetic joint infection

**DOI:** 10.1186/s13018-021-02296-7

**Published:** 2021-02-18

**Authors:** Hai Wang, Leilei Qin, Jiawei Wang, Wei Huang

**Affiliations:** 1 Department of Orthopaedics, The First Affiliated Hospital of Chongqing Medical University, Chongqing, 400016 China; 2 Department of Orthopaedics, Fuling Central Hospital of Chongqing City, Chongqing, 408099 China

**Keywords:** Periprosthetic joint infection, Synovial fluid, Inflammatory marker, Neutrophil, IL-1β

## Abstract

**Purpose:**

The purpose of this study was to investigate the role of synovial fluid interleukin (IL)-1β in diagnosing chronic periprosthetic joint infection (PJI) and to identify the optimal threshold of synovial fluid IL-1β for differentiating chronic PJI from aseptic failure after knee and hip arthroplasties.

**Methods:**

Between January 2019 and December 2019, we prospectively included patients scheduled to have a revision surgery for chronic PJI or aseptic failure after total joint arthroplasty. Then, synovial IL-1β was additionally measured along with routine preoperative diagnostic serum and synovial biomarkers. The receiver operating characteristic (ROC) curves and area under the curve (AUC) were analyzed for each biomarker to determine diagnostic efficacy.

**Results:**

Of the 93 patients included, their demographic data were not found to be statistically significant. The median synovial IL-1β levels were significantly higher in the chronic PJI group than in the aseptic group (894.73 pg/mL vs. 34.49 pg/mL, *P*<0.01). The AUC for synovial fluid IL-1β was 0.991, which was higher than serum ESR (0.627) and CRP (0.712). The optimal threshold value for detecting chronic PJI of synovial IL-1β was 312.7 pg/mL, with a sensitivity of 97.3% and a specificity of 94.64%. And the combined measurement of synovial fluid IL-1β and synovial fluid PMN% can led to a specificity of 1, and a negative predictive value (NPV) of 1.

**Conclusions:**

The present study demonstrated that synovial fluid IL-1β is a valuable biomarker for detection of chronic PJI. The combination of synovial fluid IL-1β and PMN% led to an improvement in specificity compared with evaluation of each single index.

**Trial registration:**

This study was prospectively registered on the Chinese Clinical Trial Registry (a non-profit organization, established according to both the WHO International Clinical Trials Register Platform Standard and Ottawa Group Standard), and the registering number was ChiCTR1800020440. Registered on December 29, 2018.

## Introduction

One of the most challenging complications of total joint arthroplasty (TJA) is periprosthetic joint infection (PJI), which has major health and economic consequences [1]. PJI is the leading reason for revision after total knee arthroplasty and the fourth most common reason for consultation after total hip arthroplasty [2, 3]. The distinction between PJI and aseptic prosthetic failure is critical, because the treatments for these two conditions are fundamentally different. Moreover, many cases of chronic PJI are clinically difficult to distinguish from aseptic prosthetic failure because the typical signs may be completely absent. Patients often present with chronic pain or only slight clinical symptoms. Thus, accurate diagnosis of chronic PJI plays a very important role in the overall treatment process.

The diagnosis of a PJI according to the combination of clinical manifestations, serum testing, and synovial fluid biomarkers established in the 2013 Musculoskeletal Infectious Disease Society (MSIS) criteria [4]. Although a number of markers have been shown to aid in the identification and diagnosis of PJI [5–7], including serum D-dimer [8], synovial fluid leukocyte esterase [9, 10], and synovial fluid α-defensin [11, 12], no single test is able to diagnose PJI, and multiple biomarkers are recommended [13].

Interleukin (IL)-1β, a potent pro-inflammatory cytokine, is mainly produced by activated inflammatory cells (monocytes, microglia, macrophages) [14] and may be a promising marker for chronic PJI [15, 16]. The aim of the present study was to validate the diagnostic characteristics of synovial fluid IL-1β for preoperative diagnosis of chronic PJI as either a single test or in combination with serum C-reactive protein (CRP), synovial fluid percentage of polymorphonuclear neutrophils (PMN%), or serum erythrocyte sedimentation rate (ESR) and to compare these results with the currently available diagnostic standards.

## Patients and methods

Between January 2019 and December 2019, 93 patients were enrolled in the study. All patients were scheduled to undergo revision surgery after a primary hip or knee arthroplasty, who had no prior revision or PJI history. Indications for revision surgery were chronic PJI of the hip and knee or aseptic prosthetic failure. To rule out interference from other diseases associated with elevated inflammatory markers, the following exclusion criteria were applied: (1) inflammatory arthritis such as rheumatoid arthritis or joint tuberculosis, (2) infectious diseases such as pneumonia and urinary tract infection, (3) antibiotic treatment within 2 weeks prior to surgery. All patients provided signed informed consent.

Eligible patients were assigned to the chronic PJI group or aseptic prosthetic failure group according to the 2013 MSIS criteria (Table 1). PJI was classified as chronic PJI when PJI symptoms occurred for more than 6 weeks after surgery [17]. Aseptic prosthetic failure revision was defined as single-stage revision for a reason other than infection (loosening, wear, instability, malalignment, adverse local tissue reactions, other aseptic causes) [4].

**Table 1:** The musculoskeletal society 2013 definition of PJI

MSIS definition of PJI^a^	
1	There is a sinus tract communicating with the prosthesis; or
2	Two positive periprosthetic cultures with phenotypically identical organisms; or
3	When 3 of the following 5 criteria exist: a. Elevated serum C-reactive protein (CRP) AND erythrocyte sedimentation rate (ESR) b. Elevated synovial fluid white blood cell (WBC) count OR ++change on leukocyte esterase test strip c. Elevated synovial fluid polymorphonuclear neutrophil percentage (PMN%) d. Positive histological analysis of periprosthetic tissue e. A single positive culture

^a^One of the three criteria (1, 2, or 3) must be met for diagnosis of periprosthetic joint infection

The following baseline data for the patients were recorded: age, sex, BMI, risk factors for infection (diabetes, smoking), involved joint, and time since prosthesis implantation. Blood samples were obtained after admission and analyzed for serum ESR and CRP. Synovial fluid samples were evaluated for PMN%, IL-1β, and cultures. At least three intraoperative tissue specimens were taken from patients during revision arthroplasty. These tissue samples were cultured on bovine serum-containing blood agar medium for 24 to 48 h (standard culture) and 14 days (long-term culture). Biochemical assays were performed at a biochemistry laboratory using a biology technical platform.

### Statistical analysis

Data were analyzed using SPSS version 25 software (IBM Corp., Armonk, NY). Continuous data were expressed as mean ± standard deviation, while categorical data were expressed as count and percentage. Comparisons of continuous data were performed by Student’s *t* test, while comparisons of categorical data were carried out with the chi-square test. Correlations between variables were investigated by Pearson’s correlation coefficient. Receiver-operating characteristic (ROC) curves and area under the curve (AUC) values were analyzed with MedCalc 15.2.2 software (MedCalc Software, Ostend, Belgium). Youden’s J statistic was used to determine the optimum cutoff values for the diagnosis of chronic PJI. The sensitivity, specificity, positive predictive value (PPV), negative predictive value (NPV), and accuracy were calculated for synovial fluid IL-1β and two serum markers (CRP and ESR) and evaluated. Values of *P*<0.05 were considered statistically significant.

## Results

The demographic data in the two groups are shown in Table 2. The study included a total of 93 patients, of whom 37 (39.8%) patients with infection were assigned to the chronic PJI group and 56 (60.2%) with aseptic loosening of the implant were assigned to the aseptic prosthetic failure group. The baseline characteristics in the two groups, including age, sex, BMI, and joint type, showed no significant differences. As shown in Table 3; the median synovial fluid IL-1β level in the chronic PJI group was significantly higher than that in the aseptic prosthetic failure group (894.73 pg/mL vs. 34.49 pg/mL, *P*<0.01). Median serum ESR was also significantly higher in the chronic PJI group compared with the aseptic prosthetic failure group (35.00 mm/h vs. 21.00 mm/h, *P*=0.04), as were median serum CRP (19.00 mg/L vs. 13.18 mg/L; *P*<0 .01) and synovial fluid PMN% (84.26% vs. 53.31; *P*<0.01).

**Table 2:** Demographic data for the study population

Characteristic	Infected (*N*=37)	Aseptic (*N*=56)	*P* value
Gender			0.51
Male	25(67.57%)	33(58.93%)	
Female	12(32.42%)	23(41.07%)	
Age (years)	74.57±6.01	72.15±6.54	0.08
BMI (kg/m^2^)	22.99±4.12	23.22±4.66	0.81
Joint type			0.40
Knee	20	25	
Hip	17	31	

Variables are expressed as mean ± SD or numbers (percentage)

*BMI* body mass index, *SD* standard deviation

**Table 3:** Analysis of inflammatory markers in patients with infected and aseptic revision arthroplasty

Inflammatory maker	Hip + knee		*P* value
	Infected (*n*=37)	Aseptic (*n*=56)	
ESR (mm/h)			0.04
Median	35.00	21.00	
P25, P75	(15.00, 50.00)	(12.25, 34.00)	
CRP (mg/L)			<0.01
Median	19.00	13.18	
P25, P75	(14.40, 32.80)	(5.34, 18.45)	
SF IL-1β(pg/ml)			<0.01
Median	894.73	34.49	
P25, P75	(455.91, 1779.00)	(15.70, 170.33)	
PMN %			<0.01
Median	84.26	53.31	
P25, P75	(72.57, 91.65)	(49.78, 60.11)	

*CRP* C-reactive protein, *ESR* erythrocyte sedimentation rate, *SF* synovial fluid, *PMN%*, percentage of polymorphonuclear neutrophils

**Fig. 1:**
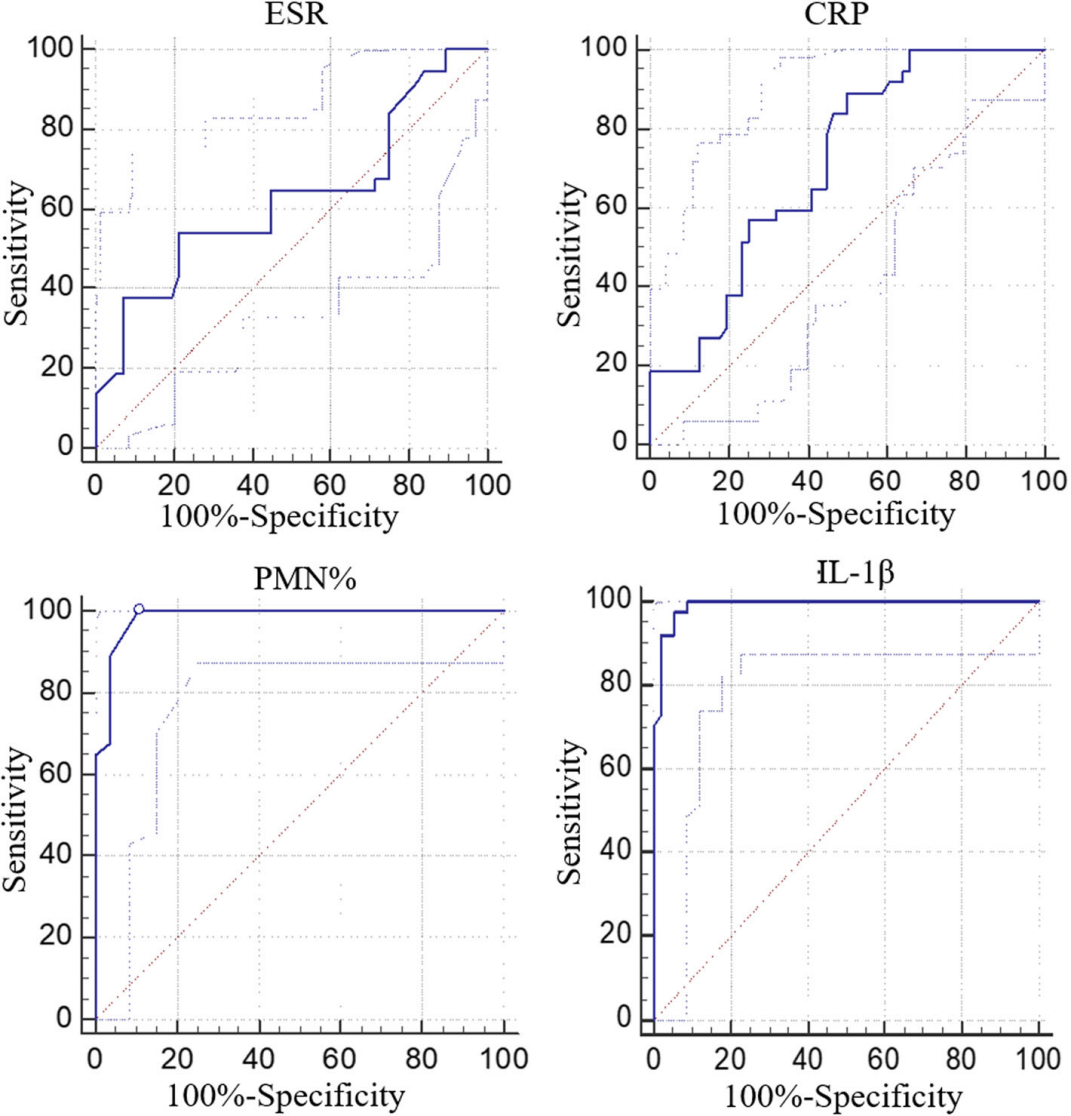
Receiver operating characteristic curves (ROCs). ROCs with the corresponding area under the curve (AUC) of various inflammatory markers of patients with PJI after TJA. SF synovial fluid, PJI periprosthetic joint infection, CRP C-reactive protein, ESR erythrocyte sedimentation rate, PMN% percentage of polymorphnuclear

ROC curves were used to measure the discriminatory strength between the chronic PJI group and the aseptic prosthetic failure group (Fig. 1). The specificity, sensitivity, and accuracy of the inflammatory markers for the diagnosis of chronic PJI were calculated, and the best cutoff values were defined using the ROC curves and associated AUC values. The AUC for synovial fluid IL-1β was 0.991 (95% CI, 0.945, 1.000) and more accurate than those for serum ESR (0.627; 95% CI, 0.521, 0.725), serum CRP (0.712; 95% CI, 0.609, 0.801), and synovial fluid PMN% (0.981; 95% CI, 0.928, 0.998).

Table 4 shows the AUC values with standard errors and 95% CIs. The synovial fluid IL-1β cutoff value of 312.7 pg/mL had sensitivity of 97.3% (95% CI, 85.8%–99.9%), specificity of 94.64% (95% CI, 85.1%–98.9%), and accuracy of 95.7% for detecting chronic PJI, with high NPV of 98.15% and high PPV of 92.31%. The sensitivity for serum CRP to detect chronic PJI was 89.19% (95% CI, 74.6%–97.0%) with specificity of 50% (95% CI, 36.3%–63.7%) and accuracy of 65.59% above a cutoff value of 13 mg/dL. Serum ESR had specificity of 78.57% (95% CI, 65.6%–88.4%) and sensitivity of 54.05% (95% CI, 36.9%–70.5%) for chronic PJI at a cutoff value of 34 mm/h. The sensitivity for synovial fluid PMN% to detect chronic PJI was 94.59% (95% CI, 81.8%–99.3%) with specificity of 92.86% (95% CI, 82.7%–98.0%) and accuracy of 93.55% above a cutoff value of 69.96%. Moreover, we evaluated the diagnostic values of combinations of these inflammatory markers for chronic PJI. The combinations of synovial fluid IL-1β and serum CRP or synovial fluid PMN% led to improvements in specificity but decreases in sensitivity. The specificity and PPV of combined synovial fluid IL-1β and serum CRP were 96.43% and 94.12%, respectively. We further found that when synovial fluid IL-1β and synovial fluid PMN% were both above their thresholds of 312.7 pg/mL and 69.96%, respectively, and they could be used to identify a positive result for chronic PJI, with sensitivity, specificity, PPV, NPV, and accuracy of 91.89%, 100%, 100%, 94.92%, and 96.77%, respectively.

**Table 4:** Sensitivity, specificity, PPV, NPV, and accuracy of inflammatory markers

Parameters	ESR (mm/h)	CRP (mg/L)	SF IL-1β (pg/ml)	SF PMN (%)	SF IL-1β +CRP	SF IL-1β+ PMN%
AUC (95%CI)	0.627 (0.521, 0.725)	0.712 (0.609, 0.801)	0.991 (0.945, 1.000)	0.981 (0.928, 0.998)	/	/
Cutoff level	34	13	312.7	69.96	SF IL-1β>312.7 +CRP>13	SF IL-1β>312.7+ PMN%>69.96
Sensitivity (%) (95%CI)	54.05 (36.9, 70.5)	89.19 (74.6, 97.0)	97.30 (85.8, 99.9)	94.59 (81.8, 99.3)	86.49 (70.43, 94.92)	91.89 (76.98, 97.88)
Specificity (%) (95%CI)	78.57 (65.6, 88.4)	50.00 (36.3, 63.7)	94.64 (85.1, 98.9)	92.86 (82.7, 98.0)	96.43 (86.62, 99.38)	100 (92.00,100)
PPV (%)	62.50	54.10	92.31	89.74	94.12	100
NPV (%)	72.13	87.50	98.15	96.30	91.53	94.92
Accuracy (%)	68.82	65.59	95.70	93.55	92.47	96.77

*CRP* C-reactive protein, *ESR* erythrocyte sedimentation rate, *CI* confidence interval, *PPV* positive predictive value, *NPV* negative predictive value

## Discussion

Chronic PJI remains the most common reason for revision after TJA, and the incidence of TJA is projected to increase sharply from 2014 to 2030 [18]. This increase will impose huge medical and economic burdens on public health [19]. Chronic PJI is often caused by microorganisms with low virulence and has considerable delays in diagnosis. It may present with atypical symptoms different from those of acute infection, which are often similar to those of aseptic loosening [20]. Patients may develop a mild systemic response and have normal laboratory markers when PJI is present as a chronic encapsulated infection [5]. For these reasons, diagnosis of chronic PJI is often confusing.

Synovial fluid biomarkers that can be used to predict PJI have been reported previously and IL-1β was included in these studies [6, 7, 15, 16]. In fact, IL-1β is a multifunctional and highly potent pro-inflammatory cytokine [21] that was confirmed to be associated with bone resorption and osteoporosis in some inflammatory diseases [22]. Nicolas et al. [23] found that IL-1β played an important role in early control of the bacterial burden in post-surgical joints. Deirmengian et al. [6] evaluated 29 PJI cases and 66 aseptic joint cases and described that the AUC for IL-1β was 0.966, with specificity of 95% (95% CI, 87%–99%) and sensitivity of 96% (95% CI, 82%,–00%). Frangiamore et al. [24] reported that the AUC, sensitivity, and specificity of IL-1β were 0.92, 90.3% (95% CI, 74%–98%), and 87% (95% CI, 76%–95%), respectively, with a decreasing trend in IL-1β between first-stage explantation and second-stage reimplantation. Gollwitzer et al. [16] found that IL-1β had sensitivity of 67% and specificity of 95% to distinguish aseptic loosening from staphylococcal infection. However, their study only focused on the diagnostic value of IL-1β for predicting PJI and did not explore the role of IL-1β in different types of PJI (acute or chronic). However, the inflammatory response in chronic PJI is very different from that in acute PJI [13]. In the present study on chronic PJI, synovial fluid IL-1β had sensitivity of 97.3% (95% CI, 85.8%–99.9%), specificity of 94.64% (95% CI, 85.1%–98.9%), and AUC of 0.991 (95% CI, 0.945–1.000) when a cutoff value of 312.7 pg/mL was used.

Elevated synovial fluid PMN% has been identified as a useful marker for diagnosis of PJI in previous studies. Trampuz et al. [25] showed that when the threshold of PMN% was >65%, the sensitivity and specificity for the diagnosis of PJI were 97% and 98%, respectively. Due to the synovial fluid composition changes with increasing postoperative time, the optimal cutoff values of PMN% for the diagnosis of acute and chronic PJI are different. During the 2013 International Consensus Meeting, the recommended cutoff value for diagnosis of acute PJI (<6 weeks after surgery) was above 90% and that for chronic PJI (>6 weeks after surgery) was above 80% [26]. Higuera et al. [27] demonstrated that the sensitivity, specificity, PPV, and NPV of PMN% above 80% for chronic hip PJI were 92.1%, 85.8%, 59.3%, and 98.0%, respectively. While in this study, when the optimal cutoff value of synovial fluid PMN% was 69.96%, the AUC, sensitivity, and specificity were 0.981 (95% CI, 0.928–0.998), 94.59% (95% CI, 81.8%–99.3%), and 92.86% (95% CI, 82.7%–98.0%), respectively.

Serum ESR and CRP have been recommended for first-line diagnostic evaluation in patients with suspected PJI by the American Academy of Orthopaedic Surgeons and the International Consensus Meeting [28]. Alijanipour et al. [29] showed that early postoperative and late chronic PJI had different thresholds of ESR and CRP, with the late chronic PJI values being higher than the early postoperative PJI values, in a retrospective review of 1962 patients who underwent revision arthroplasty for aseptic prosthetic failure (*n*=1689) or first onset of PJI (*n*=273) between 2000 and 2009. ESR >30 mm/h and CRP >10 mg/L were recommended as the optimal thresholds for diagnosis of chronic PJI in the 2013 International Consensus Meeting on Surgical Site and Periprosthetic Joint Infection [30]. In our study on chronic PJI, similar results were obtained again, and the optimal cutoff values of serum ESR were 34 mm/h with specificity of 78.57% and sensitivity of 54.05% indicating the limited diagnostic value for chronic PJI. In the same way, the sensitivity and specificity of serum CRP were 89.19% (95% CI, 74.6%–97.0%) and 50% (95% CI, 36.3%–63.7%) for the diagnosis of chronic PJI with a cutoff value of 13 mg/dL.

A single indicator cannot provide 100% diagnostic accuracy and in the presence of high clinical suspicion, a combination of tests should be used to refute or confirm the possibility of infection [31]. The present study explored diagnosis of chronic PJI using combinations of synovial fluid IL-1β and serum CRP or synovial fluid PMN%. When synovial fluid IL-1β was combined with serum CRP or synovial fluid PMN%, the specificity was 96.43% or 100%, respectively, while the sensitivity was decreased. This trade-off is typical for many tests. After comparing the two combinations, we found that the combination of synovial fluid IL-1β and PMN% had the highest accuracy for detecting chronic PJI. Furthermore, when both synovial fluid IL-1β and PMN% were above their thresholds of 312.7 pg/mL and 69.96%, respectively, the specificity, PPV, and accuracy reached 100%, 100%, and 96.77%, respectively. These results indicate that the combination is more accurate than each single index alone.

## Conclusions

In this study, synovial fluid IL-1β was considered a specific molecular marker for the diagnosis of chronic PJI and its optimal cutoff value was established at 312.7 pg/mL. Compared with serum ESR and CRP, the sensitivity and specificity of synovial fluid IL-1β were higher for distinguishing aseptic prosthetic failure after TJA from chronic PJI. However, application of synovial fluid IL-1β to predict the diagnosis of chronic PJI is relatively rare at present, and more evidence is needed to support the diagnostic value of synovial fluid IL-1β.

## Abbreviations

PJI: Periprosthetic joint infection
IL: Interleukin
MSIS: Musculoskeletal Infectious Disease Society
ROC: Receiver operating characteristic
AUC: Under the curve
NPV: Negative predictive value
PPV: Positive predictive value
TJA: Total joint arthroplasty
CRP: C-reactive protein
ESR: Erythrocyte sedimentation rate
SF: Synovial fluid
PMN%: Percentage of polymorphonuclear neutrophils
